# Chromatin loop anchors contain core structural components of the gene expression machinery in maize

**DOI:** 10.1186/s12864-020-07324-0

**Published:** 2021-01-06

**Authors:** Stéphane Deschamps, John A. Crow, Nadia Chaidir, Brooke Peterson-Burch, Sunil Kumar, Haining Lin, Gina Zastrow-Hayes, Gregory D. May

**Affiliations:** 1 Corteva Agriscience, 8325 NW, 62nd Avenue, Johnston, Iowa, 50131 USA; 2 Corteva Agriscience, The V-Acendas, Atria Block, 12th Floor, Plot No.17, Madhapur, Hyderabad, Telangana 500081 India

**Keywords:** Maize, Chromatin, Loop, Anchor, TAD, Domain, Hi-C, RNA-Seq, ATAC-Seq

## Abstract

**Background:**

Three-dimensional chromatin loop structures connect regulatory elements to their target genes in regions known as anchors. In complex plant genomes, such as maize, it has been proposed that loops span heterochromatic regions marked by higher repeat content, but little is known on their spatial organization and genome-wide occurrence in relation to transcriptional activity.

**Results:**

Here, ultra-deep Hi-C sequencing of maize B73 leaf tissue was combined with gene expression and open chromatin sequencing for chromatin loop discovery and correlation with hierarchical topologically-associating domains (TADs) and transcriptional activity. A majority of all anchors are shared between multiple loops from previous public maize high-resolution interactome datasets, suggesting a highly dynamic environment, with a conserved set of anchors involved in multiple interaction networks. Chromatin loop interiors are marked by higher repeat contents than the anchors flanking them. A small fraction of high-resolution interaction anchors, fully embedded in larger chromatin loops, co-locate with active genes and putative protein-binding sites. Combinatorial analyses indicate that all anchors studied here co-locate with at least 81.5% of expressed genes and 74% of open chromatin regions. Approximately 38% of all Hi-C chromatin loops are fully embedded within hierarchical TAD-like domains, while the remaining ones share anchors with domain boundaries or with distinct domains. Those various loop types exhibit specific patterns of overlap for open chromatin regions and expressed genes, but no apparent pattern of gene expression. In addition, up to 63% of all unique variants derived from a prior public maize eQTL dataset overlap with Hi-C loop anchors. Anchor annotation suggests that < 7% of all loops detected here are potentially devoid of any genes or regulatory elements. The overall organization of chromatin loop anchors in the maize genome suggest a loop modeling system hypothesized to resemble phase separation of repeat-rich regions.

**Conclusions:**

Sets of conserved chromatin loop anchors mapping to hierarchical domains contains core structural components of the gene expression machinery in maize. The data presented here will be a useful reference to further investigate their function in regard to the formation of transcriptional complexes and the regulation of transcriptional activity in the maize genome.

**Supplementary Information:**

The online version contains supplementary material available at 10.1186/s12864-020-07324-0.

## Background

Genomic DNA, the largest molecule in a cell, is packed with histone to form chromatin. Recent improvements in the molecular characterization of chromatin have shown that its spatial structure can be dissected into separate functional domains, ranging in sizes from a few Kbps to Mbps [1–3]. Those domains include A and B compartments, known to be associated with the euchromatic and heterochromatic portions of a genome, respectively. Other domains, known as Topologically-Associating Domains (“TADs”) have now been discovered in multiple organisms, including humans [4], animals [5] and plants [6]. Other features, known as “chromatin loops”, are thought to be critical factors for the spatial regulation of gene expression through a loop extrusion mechanism allowing the three-dimensional positioning of distal regulatory elements, and their interaction with proximal elements regulating the expression of specific genes [7].

In plants, the understanding of chromatin organization is mainly derived from a relatively small number of studies in species that include Arabidopsis [6], rice [8] and maize [9]. Large plant genomes can be partitioned into TAD-like domains, which are in fact compartment domains [9]. Some domains are enriched in active genes, open chromatin and active histone marks while others are enriched in epigenetics signatures typical of repressive domains (including DNA methylation) [9]. In maize, chromatin loops can be formed between active chromatin domains [9], forming a rich and complex molecular interaction network linking distal and proximal regulatory elements [10], suggesting that the presence of chromatin loops in repeat-rich plant genomes could be a mechanism allowing distal regulatory elements to activate, or repress, genes separated from those elements by condensed heterochromatin [11]. Specific variants in regulatory elements also may contribute to variations in gene expression through long-range chromatin interactions, linking eQTLs to their associated genes via chromatin loop interactions [12].

While long-range loop formation between chromatin domains has been shown to link together gene-rich and distal regulatory regions in complex plant genomes, no study yet has been performed to determine the extent of such mechanism and its genome-wide correlation to TAD-like domains and gene transcription in maize. In addition, while Hi-C is known as a “low-resolution” method capturing mainly long-range chromatin loops [9], its relationship to higher resolution loops detected with methods such as Chromatin Interaction Analysis by Paired-End Tag Sequencing (“ChIA-PET”) [10] or HiChIP [11] still remains to be determined, along with the predisposition of expressed genes and regulatory elements to co-locate with specific loop types. In this study, the overall structure of chromatin loops in maize and their prevalence as a putative mode of action associated with the regulation of gene transcription were evaluated. Structural relationships between TAD-like domains, chromatin loops, gene expression and open chromatin regions (used as indirect signals for protein binding to DNA) were systematically assessed through ultra-deep sequencing of Hi-C libraries, combined with the generation of RNA-Seq and Assay for Transposase-Accessible Chromatin using sequencing (“ATAC-Seq”) datasets from the same maize tissue, and further compared to public maize high-resolution interactome and eQTL functional datasets [10, 13]. Results showed substantial overlaps between those features, revealing chromatin loops as biological components of the gene regulation machinery in maize, with a restricted number of chromatin loop anchors as its core structural unit.

## Results

Whole maize B73 leaf tissue was collected at development stage v04. The same batch of plants, at the same stage (v04) and divided into four biological replicates (four plants per replicate) was used for gene expression profiling (RNA-Seq; four replicates), three-dimensional chromatin profiling (Hi-C; two replicates) and accessible chromatin (ATAC-Seq; three replicates).

The ultra-deep sequencing of two Hi-C biological replicates led to a total number of 3,435,596,872 and 3,424,795,714 raw paired reads, for replicates 1 and 2, respectively. Filtering of the raw data and mapping to the B73 AGPv4 reference genome sequence led to the detection of 392,396,275 and 449,828,472 Hi-C contact pairs, after combining inter-chromosomal and intra-chromosomal contacts for replicates 1 and 2, respectively (Table 1).

**Table 1:** **Interaction metrics for the chromosomal distribution of Hi-C contact pairs**

	Inter-chromosomal Contacts	Intra-chromosomal Contacts	Intra-chromosomal Short-Range (<20Kbps) Contacts	Intra-chromosomal Long-Range (>20Kbps) Contacts
Replicate 1	92,042,539	300,353,736	215,014,034	85,331,655
Replicate 2	95,674,415	354,154,057	252,256,720	101,886,819

Intra-chromosomal contacts are further divided into short range (<20Kbps) and long-range (>20Kbps) contacts.

**Fig. 1:**
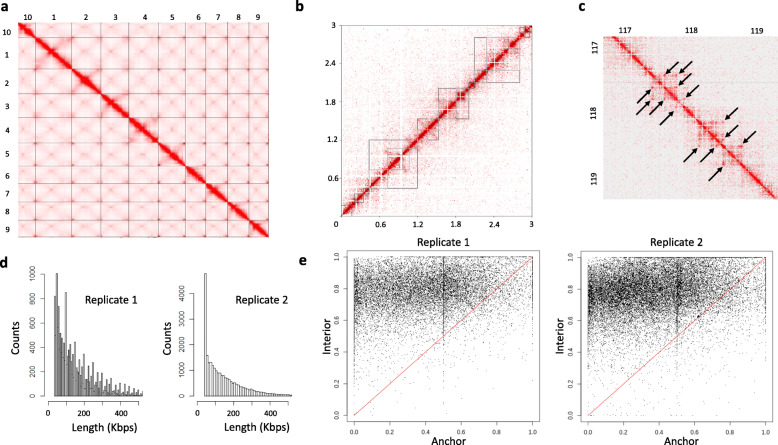
Interaction matrices of maize leaf v04 Hi-C replicate 1 library. **a** Genome-wide interaction matrix. Each interaction is represented by a “pixel” on the map and the frequency of interactions within a particular region is proportional to the number of pixels. Chromosomes are labeled by numbers (1 to 10, starting with Chr10 at the top left). **b** Interaction matrix for Chr01. TAD-like chromatin domains and nested sub-domains are marked by squared areas indicating a higher frequency of interactions within a particular chromosomal region. Axis labels indicate coordinates in Mbps. **c** Interaction matrix for a ~ 3Mbps region located on Chr03. The Hi-C contact matrix shows evidence of domains, with increased cis-interactions at their borders, suggesting the formation of chromatin loops. Seven chromatin loops, marked by solid arrows on both side of the diagonal, are shown as examples. **d** Distribution of chromatin loop lengths (e.g., distance between anchors) for replicate 1 and replicate 2 Hi-C datasets. Lengths are shown in Kbps (x-axis). The origin of the size oscillation pattern shown for replicate 1 remains to be determined. **e** Repeat density analysis in anchor and loop interior regions for all loops detected in replicate 1 and replicate 2. Each dot in the graph represents an individual loop. X-axis: fraction of bases within whole anchor regions (0 to 1) that are occupied by conserved elements; Y-axis: fraction of bases within whole interior regions (0 to 1) that are occupied by conserved elements

Hi-C interaction matrices showed strong interactions between neighboring loci on euchromatic arms, accompanied by cis and trans interactions between centromeric and telomeric regions and cis interactions between chromosomal arms (Fig. 1a). Further analysis showed evidence of hierarchical TAD-like domains [14], covering most of the maize genome in a nested fashion (Table 2) (Fig. 1b). A total of 17,978 and 18,739 domains were detected for Hi-C replicates 1 (See Additional File 1) and 2 (See Additional File 2), respectively. Here, large “level 0” domains contain series of nested “sub-domains” (level 1 to 6) representing approximately 60% of all detected domains (Table 2). Previous studies have shown that the rice genome contained extensive hierarchical chromatin interactions [8]. Others have shown that TAD-like domains in larger plant genomes such as maize are essentially compartment domains [9], where transcriptionally active domains are separated by large inactive heterochromatic domains. The results shown here suggest a model where “level 0” TAD-like compartment domains may contain multiple nested layers of chromatin interactions, marked by the presence of smaller nested “sub-domains”.

**Table 2:** Hierarchical TAD-like domain counts for replicates 1 and 2

TAD level	Replicate 1 100% read count	Replicate 1 50% read count	Replicate 2 100% read count	Replicate 2 50% read count
0	7131	7989	7257	8028
1	6621	4982	6997	6231
2	3206	2084	3384	2717
3	835	428	911	603
4	157	57	165	83
5	24	10	25	10
6	4	0	0	0

Hierarchical domains (level 0 to 6) were detected in replicates 1 and 2 from Hi-C contact maps generated using 100 and 50% total sequencing read counts. Domains (level 0) were further divided into nested “sub-domains” (levels 1 to 6).

To determine whether ultra-deep sequencing of each Hi-C biological replicate impacted hierarchical TAD-like domain detection and resolution, random Hi-C sequencing read datasets representing 50% of all total sequencing reads were generated to create new Hi-C interaction matrices. The new matrices then were used to compute new domains for each Hi-C replicate, which were subsequently compared to the ones computed with full read counts (Table 2). While the total number of domains did not significantly improve with higher initial read counts, it led nonetheless to the dissection of some domains into additional sub-domains.

In addition to TAD-like domains, the chromosomes could be partitioned into larger chromosomal A/B compartments by eigenvector analysis of the Hi-C interaction matrices. In maize, those compartments would divide each chromosome into two global A compartments at the chromosomal ends, flanking a global B compartment, corresponding roughly to its pericentromeric heterochromatic regions [9]. Eigenvector analysis of chromosome 1 at 500Kb resolution, using Hi-C interaction matrices generated from the “50% read count” and “100% read count” datasets shown above, led to the detection of global compartments similar in structure to what had been already published [9] and between each other, therefore indicating that, contrary to domain detection, increasing read counts did not significantly improve compartment detection.

Compartments, domains and sub-domains are prominent organizational features in the maize genome. Increased interactions at their borders suggested the existence of chromatin loops (Fig. 1c). A total of 17,176 and 25,917 chromatin loops were initially detected for Hi-C replicates 1 (See Additional File 3) and 2 (See Additional File 4), respectively. To confirm their validity, HICCUPS analyses were run at various resolutions from interaction matrices generated with distinct read counts (Table 3). Expectedly, loop counts varied with both read counts and resolution of detection. Based on those results, it was determined that the initial datasets for both replicates provided an appropriate number of high-resolution loops for subsequent analysis.

**Table 3:** Chromatin loop counts at various resolutions and read counts

Loop detection resolution	Replicate 2 100% read count	Replicate 2 50% read count
5Kbps	16,568	9118
10Kbps	25,917	16,950
25Kbps	18,397	13,697
50Kbps	35,832	24,358

Chromatin loops were detected with the HICCUPS software tool, from Hi-C interaction matrices generated using 100 and 50% total sequencing read counts.

Distances between anchors within a loop varied from 30Kbps to >1Mbp (Fig. 1d). Interestingly, repeat element density analysis, between anchors and regions located between two anchors, labeled as “loop interiors”, showed that repeats were more prevalent in loop interiors (Fig. 1e). There were 7917 loops present in both replicates, with both anchors overlapping by at least 1 bp, and only 1268 loops in replicate 1 and 2657 loops in replicate 2 where none of the anchor overlap, indicating that a significant fraction of the loops from both replicates shared one anchor only.

Additional comparisons were made with prior sets of high-resolution chromatin interactions detected with the HiChIP [11] and ChIA-PET methodologies in B73 [10]. The HiChIP data were generated using antibodies targeting histone modifications associated with transcriptional activations (H3K4me3) and repression (H3K27me3) while the ChIA-PET data were generated using antibodies targeting histone modifications associated with transcriptional activation only (H3K4me3 and H3K27ac) and filtered into a final chromatin interaction dataset (see Supplementary Data 16 in [10]). Those high-resolution datasets were expected to capture local interactions (such as interactions between regulatory elements), typically not detectable via Hi-C sequencing. Co-location analysis of replicate 2 loop anchor regions with high-resolution interaction loop anchors (Table 4) showed that, while ~ 9 to 18% of high-resolution interaction datasets fully overlapped with Hi-C loops (“2 co-located anchors”), a majority (~ 68 to 80%) of all remaining high-resolution loops shared one anchor with replicate 2 Hi-C loops.

**Table 4:** Co-location of loop anchors with high-resolution conformation capture datasets

	2 co-located anchors	1 co-located anchor	0 co-located anchor	Total loop counts
ChIA-PET (chromatin interactions)	4511	15,890	3817	24,218
HiChIP (H3K27me3)	3612	24,909	11,297	39,818
HiChIP (H3K4me3)	10,021	45,011	11,980	67,012

High-resolution ChIA-PET and HiChiP interaction coordinates were compared to replicate 2 Hi-C loop anchor coordinates. Co-location was determined with anchors exhibiting at least 50% overlap between the two data types. Antibodies targeting histone modifications in HiChIP are shown. High-resolution loops were counted once only, after prioritization, in the following order, for exhibiting 2, 1 or 0 co-located anchors.

**Fig. 2:**
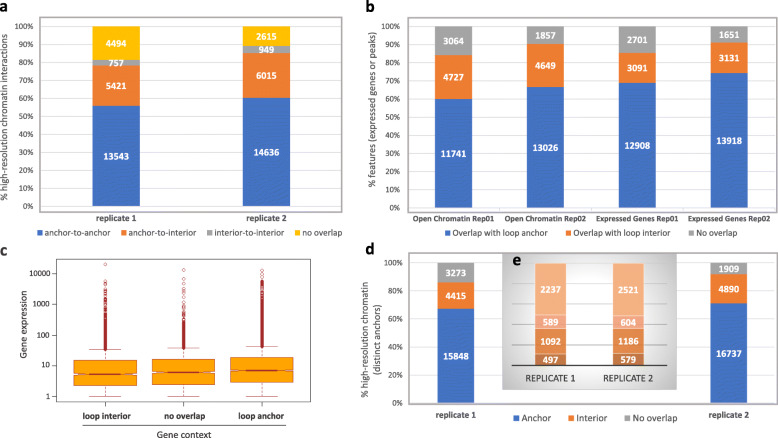
Characterization of Hi-C chromatin loop anchor and interior regions. **a** Overlap of replicates 1 and 2 chromatin loops with high-resolution chromatin interactions (see text). Percentages of high-resolution chromatin interactions (Y-axis) mapping with replicates 1 and 2 chromatin loops are shown (anchor-to-anchor, anchor-to-interior, interior-to-interior or not mapping). Numbers within each box indicate counts of high-resolution chromatin interactions for each category. **b** Overlap of B73 leaf v04 ATAC-Seq peaks and expressed genes (see text) with replicates 1 and 2 chromatin loops. Percentages of peaks and expressed genes overlapping with loop anchors, loop interiors, or not overlapping, are shown (Y-axis). Numbers within each box indicate counts of peaks or expressed genes for each category. **c** Wilcoxon test plots for gene expression differentials between expressed genes overlapping with replicate 1 loop anchors, overlapping with replicate 1 loop interiors or not overlapping. **d** Overlap of distinct high-resolution chromatin interaction anchors with Hi-C chromatin loop anchors and interiors. Percentages (Y-axis) of distinct high-resolution anchors mapping to Hi-C loop anchors, interiors, or not overlapping to any Hi-C features are shown for replicates 1 and 2 chromatin loops (respective counts are shown within each box). **e** High-resolution anchors co-locating with replicates 1 and 2 loop interiors and, from bottom to top, 1) expressed genes flanked by overlapping open chromatin peaks or peaks located <2Kbps away; 2) expressed genes and open chromatin peaks located >2Kbps away from the gene; 3) open chromatin peaks and expressed gene located >2Kbps away from the peak; and 4) no overlapping features (expressed gene or open chromatin peak). Counts are shown for each category

Further analysis comparing replicates 1 and 2 Hi-C datasets with high-resolution ChIA-PET maize data indicated that both anchors from up to 60% of all high-resolution interactions overlapped with anchors derived from Hi-C replicates 1 or 2 (Fig. 2a). Up to 28% of the remaining high-resolution interactions shared one anchor with loop interior regions, while a small number of interior regions overlap fully with both high-resolution anchors. Interestingly, only 23,536 out of the 48,430 anchors forming high-resolution chromatin interactions were deemed as distinct, based on their exact physical coordinates.

A similar analysis was performed with a prior low-resolution Hi-C dataset [9], where 96% of all low-resolution Hi-C loops (5393 out of 5616) shared either one or two anchors with the replicate 2 Hi-C loop dataset. Taken together, these results suggested that deep sequencing of Hi-C libraries captured specific regions of the maize genome that were involved in transcriptional regulation. In addition, the repeated detection of loops sharing one anchor between multiple independent datasets suggested a regulatory environment where a conserved set of genomics regions was involved in complex interaction networks regulating multiple genes through the formation of distinct loops.

Another co-location analysis was performed where Hi-C loop anchor coordinates were compared for each replicate to their respective hierarchical TAD-like domain boundary coordinates. In here, loop spans (excluding loops detected in AGPv4 Chr0), rather than individual loop anchors, were analyzed and counted, depending on whether loops were fully embedded within a hierarchical TAD-like domain, or whether one or both loop anchors were located within 2Kbps flanking domain boundaries. Results are shown in Table 5 (replicate 1) and Table 6 (replicate 2).

**Table 5:** Replicate 1 loop overlap with hierarchical TAD-like domains

	Embedded loops	1 co-located anchor	2 co-located anchors
Level 0	4272	1214	85
Level 1	2484	1024	103
Level 2	852	366	67
Level 3	185	76	7
Level 4	18	13	0
Level 5	2	3	0
Level 6	2	1	0

Out of a total of 16,863 loops (excluding Chr0 loops), 10,774 overlap with replicate 1 domains, including 7815 fully embedded within domains (“Embedded loops”). Hierarchical TAD-like domains (levels 0 to 6) are shown on the left column. “Embedded loops”: Hi-C loops are fully included within a domain; “1 co-located anchor”: one Hi-C loop anchor overlaps with one of the two domain boundaries only, while the other anchor is located inside the domain; “2 co-located anchors”: both Hi-C loop anchors overlap with boundaries from the same domain. Loops overlapping with multiple Level 0 domains or not overlapping with any domains are not shown.

**Table 6:** Replicate 2 loop overlap with hierarchical TAD-like domains

	Embedded loops	1 co-located anchor	2 co-located anchors
Level 0	5360	2484	308
Level 1	3117	2104	371
Level 2	1109	769	142
Level 3	213	164	30
Level 4	35	19	4
Level 5	3	4	0

Out of a total of 25,628 loops (excluding Chr0 loops), 16,236 overlap with replicate 2 domains. Definitions are similar to the ones described on Table 5.

Approximately 37% of all loops detected from Hi-C replicates 1 and 2 either co-located with multiple Level 0 domains (i.e., each anchor was located in a distinct domain) or did not overlap with any domain. At least 60% of the remaining loops as shown on Tables 5 and 6 were fully embedded within a single domain.

Sequence coverage peaks from ATAC-Seq libraries are generally seen as a proxy for transcription factor binding sites and gene regulatory elements in genomic DNA [15]. Sequencing of three ATAC-Seq biological replicates led to the detection of 20,955 to 39,584 open chromatin peaks (see Additional file 5). For each replicate, peaks were classified as located between two nucleosomes (“nucleosome-free” or “NF”) or overlapping one or more nucleosome (“multi-nucleosome” or “MN”), based on the distance between ATAC-Seq paired sequencing reads. While NF peaks tended to be discrete peaks (~ 100 bps) located immediately upstream or downstream of genes (proximal peaks), or in intergenic regions (distal peaks), MN peaks tended to be broad peaks primarily centered over entire gene regions. A list of 32,009 “consensus” NF and MN peaks was generated, where a peak had to be present in at least two individual replicates to be conserved, out of which only the 19,532 NF peaks were kept for further analysis (See Additional File 6).

Co-location analysis of ATAC-Seq NF peaks with chromatin loops initially was performed using whole replicate 1 and 2 Hi-C loop datasets and assessed, based on the following criteria. As many chromatin loop anchors were shared between multiple loops, in a significant number of cases, a peak could align to an anchor for one loop and a loop interior for another loop. Therefore, peak overlaps to loop regions were determined first based on their potential overlap to at least one loop anchor. If no overlap was detected, peaks were assessed based on their potential overlaps to loop interiors, then to genomic regions located outside of chromatin loops. Using this approach, up to 13,026 peaks, out of 19,532, overlapped primarily with anchors, while up to 4649 peaks overlapped primarily with loop interiors (Fig. 2b). On the other hand, only 33% of all anchors overlapped with open chromatin peaks, suggesting technical constraints that limited the total number of peaks detected here, but also the possibility for distinct functions, or lack thereof, for some anchors not overlapping with peaks.

A similar outcome was observed for expressed genes. The total number of genes in B73 was estimated to be 38,847, out of which 18,700 were defined as “expressed” (see Methods) in B73 leaf whole tissue (see Additional file 7). Of these, up to 13,918 (74.4%) primarily overlapped with chromatin loop anchors (Fig. 2b). When adding expressed genes overlapping with loop interiors, up to 91.1% of all expressed genes in leaf overlapped with chromatin loops. Among the remaining 20,147 un-expressed genes, 8162 overlapped primarily with loop anchors (from replicate 1) while another 7672 overlapped with loop interiors, suggesting that silenced genes also could be regulated through loop formation. No major differences in expression levels were observed between genes overlapping with loop anchors, genes overlapping with loop interiors and genes located outside of loops (Fig. 2c; replicate 1 only).

The 23,536 distinct anchors from high-resolution chromatin interactions were mapped to determine whether peaks and expressed genes overlapping with Hi-C loop interiors (Fig. 2d) also could overlap with high-resolution loop anchors. Up to 92% of distinct high-resolution anchors were contained within Hi-C chromatin loops, including up to 4890 overlapping with Hi-C loop interiors (Fig. 2d). Among those, 49% overlapped with at least one expressed gene or an open chromatin peak (Fig. 2e). Conversely, 42.7% of expressed genes and 29.9% of open chromatin peaks present in replicate 2 loop interiors (Fig. 2b) also overlap with high-resolution loop anchors.

A total of 50,929 eQTLs associated to over 18,000 maize genes, derived from genotyping-by-sequencing, high density arrays and RNA-Seq data (see Supplemental Table 6 in [13]) were aligned to anchors. For replicate 2, 17,020 eQTLs had the lead SNPs and expression traits located on the same anchor while 2632 had them located on separate anchors from the same loop (for replicate 1, those numbers were 10,938 and 1829, respectively). Co-location occurred on 8745 distinct replicate 2 anchors and 8907 distinct replicate 1 anchors. Out of the 43,398 unique SNPs derived from the eQTL dataset, 25,162 and 27,252 overlapped with replicate 1 and replicate 2 anchors, respectively. Interestingly, 29,248 eQTL SNPs also overlapped with the high-resolution chromatin interactions described above [10].

**Fig. 3:**
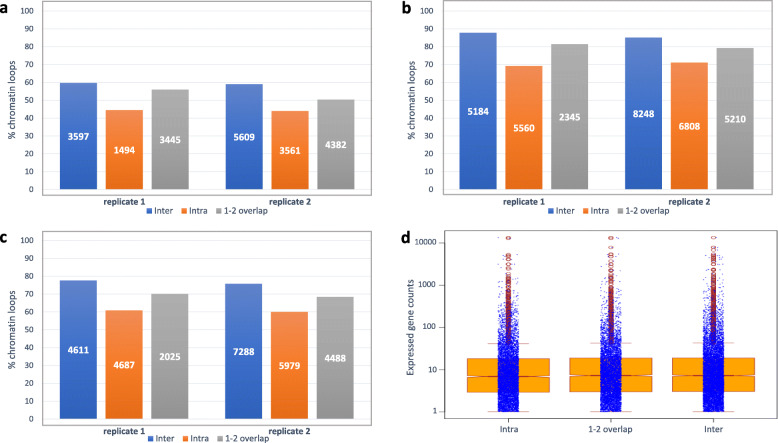
Domain-dependent co-location analysis of Hi-C chromatin loops with expression features (open chromatin, expressed genes and eQTL traits) overlapping with at least one of their anchors (**a**-**c**). Domain-dependent correlation analysis with gene expression (**d**). “Inter”: loop span multiple domains with anchors located in distinct domains; “Intra”: loops are fully embedded within a single domain or sub-domain; “1–2 overlap”: loops are contained within one domain or sub-domain, with one or both anchors overlapping with domain or sub-domain boundaries. Percentages of loops from each type co-locating with expression features are shown on the Y-axis. Respective absolute counts are listed within each box. **a** Co-location of Hi-C replicate 1 and 2 chromatin loops in relation to their overlap with open chromatin regions. **b** Co--location of Hi-C replicate 1 and 2 chromatin loops in relation to their overlap with expressed genes. **c** Co--location of Hi-C replicate 1 and 2 chromatin loops in relation to their overlap with eQTL-associated traits. **d** Gene overlap with chromatin loops and domain types are shown for Hi-C replicate 2 only. (Y-axis) Gene expression levels (computed by averaging TPM counts for four biological replicates). Mean TPM counts: 35.121 (Intra); 37.567 (1–2 overlap); 38.189 (Inter)

To assess whether ultra-deep sequencing of Hi-C libraries captured chromatin loops carrying distinct functions, loops mapping to hierarchical TAD-like domains (as described in Tables 5 and 6) were further analyzed by determining the overlaps of their anchor regions (at least 1 bp overlap) with the same ATAC-Seq NF peaks, expressed genes and eQTL expression trait datasets used above (Fig. 3). Anchors from loops mapping to distinct domains or overlapping with one or two boundaries (at least 2Kbps overlap) within the same domain intersected more frequently with ATAC-Seq NF peaks (Fig. 3a), expressed genes (Fig. 3b) and eQTL expression traits (Fig. 3c), than anchors from fully embedded loops. The different frequencies (shown here in percentage points) were consistent between Hi-C replicates 1 and 2 (Fig. 3) and could reflect domain types with different organizational features or associated with distinctive biological functions [9]. No apparent differences were observed when plotting expression levels for genes overlapping with replicate 2 loop anchors in regard to the domain types they mapped to (Fig. 3d), suggesting that expression patterns and regulation of genes mapping to chromatin loops within domains did not necessarily differ from the ones located in loops overlapping domains or mapping to domain boundaries. Further studies, focusing for example on specific epigenetic marks or patterns in genes of interest, might be required to better understand potential variations in the mechanisms establishing their expression and regulation.

To further explore potential relationships between gene expression and loop formation, gene content of anchors overlapping with at least one NF peak was assessed. In this analysis, anchors were annotated based on the activity of overlapping genes located next to NF peaks. The data show that 62% of all NF peaks overlapping with chromatin loop anchors either overlapped or were located <2Kbps away from expressed genes, suggesting those peaks may correspond to proximal regulatory regions, including promoters. Conversely, analysis of gene activity in loop anchors (including genes present in multiple anchors) showed that, for 71% of anchors harboring genes, at least one of those genes was expressed (See Additional File 8). Expression was associated with the absence of inactive genes within the anchor for 88% of those anchors, and this trend was generally accentuated by the presence of proximal open chromatin peaks. 74% of the remaining anchors carried distal peaks, possibly representing long-range regulatory elements.

Anchor annotation using replicate 1 as an example (See Additional File 9) showed that, of all 18,296 anchors with at least one expressed gene, 15,058, or 82%, had only one expressed gene. A total of 3684 anchors contained at least one peak and no expressed genes (including 2285 containing one peak only). Out of those, 2897 were part of a loop where the opposite anchor contained at least one expressed gene and at least one peak, suggesting functional interactions between proximal and distal elements facilitated by loop formation. In addition, there were a total of 5488 loops containing at least one expressed gene in both anchors, including 1058 harboring at least one peak in each anchor, indicating potential interactions between proximal elements. Interestingly, out of the 11,066 anchors from replicate 1 with no known peak or expressed gene, only 2368 were combined together into one chromatin loop, suggesting that only 6.8% (1184/17,176) of all long-range chromatin loops from replicate 1 are potentially devoid of detected gene expression activity or open chromatin regions.

## Discussion

A majority of expressed genes in leaf tissue and discrete open chromatin regions (located primarily at the 5′ and 3′ ends of genes, as well as other intergenic regions that may correspond to proximal and distal regulatory element binding sites) co-locate with a conserved set of chromatin loop anchors. Chromatin loops co-locate with hierarchical TAD-like domains and sub-domains, where chromatin interactions may occur in a series of nested hierarchical sub-domains contained within larger compartment domains. Based on their co-location, loops can be divided into specific categories, each reflecting apparent variable overlap frequencies with open chromatin regions, expressed genes and public eQTL associated traits. This suggests the possibility of distinct loop types, and, possibly, distinct molecular functions, although no apparent differences in gene expression was observed between genes mapping to various loop types and regions. A vast majority of the chromatin loops detected via Hi-C share anchors with other loops, including high-resolution interactions detected in the same genotype (B73), but with more accurate techniques (HiChIP and ChIA-PET). A subset of these anchors overlaps with one or more expressed genes and open chromatin regions. Many are shared between multiple loops, including some that are fully embedded within larger ones. Taken together, these data indicate that loop formation may be linked to gene transcription and regulation through highly dynamic three-dimensional mechanisms reminiscent of enhancer/promoter interaction complexes described elsewhere [10] and involving a conserved set of genomic regions.

Interestingly, while most of the detected active genes and open chromatin regions co-locate with chromatin loop anchors, a large portion of anchors, conversely, do not overlap with any genes or ATAC-seq peaks. It is likely that, due to technical constraints, the 19,532 consensus NF peaks used here represent a conservative estimate of the total peak counts. Therefore, many of the “empty” loop anchors listed here may harbor putative open chromatin regions that were undetected in the present study.

When combined with the observation that repeat content is denser in loop interiors than in the flanking loop anchors, the results shown here are in line with a phase separation model of chromatin dynamics in maize [16]. In this model, chromatin compartmentalization in transcribed regions is marked by the exclusion of adjacent repeat-rich regions and the formation of supramolecular condensates driving the regulation of gene expression through the active interactions between molecules that include proteins and nucleic acids. Such interaction potentially could be marked by the presence of ATAC-Seq peaks in loop anchors. Some of the loops analyzed here exhibit multiple peaks in their anchors (see Additional file 9), suggesting the possibility of multi-protein assemblies in the vicinity of potential DNA binding sites. As the work done here was performed on whole tissue, it is possible that some of the signals observed are actually due to specific cell types. As a result, more cell type-specific studies will be required to assess the mechanistic aspects of tri-dimensional regulation of gene transcription in maize. Of interest will be the need to determine whether loop formation facilitates gene expression or instead derives from it.

## Conclusions

The results shown here indicate that the scope of the functional maize genome may be narrowed down to a restricted number of loop anchors. Data suggest that these regions anchor regulatory complexes interacting with a majority of those genes being expressed in maize, through three-dimensional interactions hypothesized to be consistent with a phase separation model of chromatin dynamics. While further investigation will be required to confirm this model, the various sequencing datasets generated for this study will facilitate the systematic discovery of new motifs, such as enhancers and eQTL variants, making them prime candidates for future functional genomics studies through their direct association with the transcription of specific gene sets. In addition, the current datasets may be used to facilitate prediction modeling for breeding and genomic selection applications, through the ranking of specific variants in loop anchors and of their predicted functional impact in genotypes of interest.

## Methods

### Plant material

Whole maize B73 v04 leaf tissue, grown in a greenhouse, was collected and stored at − 80 °C after grinding. Plants were divided into four biological replicates (four plants per replicate). The same replicates were used for gene expression profiling (RNA-Seq), accessible chromatin (ATAC-Seq), and three-dimensional chromatin profiling (Hi-C).

### Hi-C library construction

Grinded tissue from two biological replicates (replicates 1 and 2), kept at − 80 °C and each containing v04 leaves from four plants, were used for preparing two Hi-C replicate libraries. Libraries were constructed with the Dovetail Hi-C Library preparation Kit (Dovetail), according to the manufacturer’s instructions, including protocol modifications recommended by Dovetail for plant tissue. Since tissues had previously been grinded (in liquid nitrogen) before long-term storage at − 80 °C, 2 ml 1X PBS and 81 μl 37% formaldehyde were added directly to 250 mg of frozen and grinded tissue in a 15-ml tube then incubated at room temperature for 15 min, prior to completing the protocol.

### RNA-Seq library construction

Total RNA was extracted from grinded v04 leaf tissue previously stored at − 80 °C using the RNeasy 96 kit (Qiagen). Four biological replicates, each containing v04 leaves from four plants, were used for total RNA extraction and subsequent poly(A) RNA library construction. Poly (A) RNA-Seq libraries were built using the TruSeq stranded mRNA library preparation kit (Illumina) according to the manufacturer’s instructions.

### ATAC-Seq library construction

Grinded tissue from three biological replicates, kept at − 80 °C and each containing v04 leaves from four plants, were used for nuclei extraction and ATAC-Seq library preparation. Prior to library construction, 1 L of 1X Nuclei Isolation Buffer (NIB) solution (10 mM Tris-HCl, pH 8.0; 10 mM EDTA pH 8.0; 7.45 g/L KCl; 171.2 g/L sucrose; 1 g/L spermidine trihydrochloride; 0.35 g/L spermine tetrahydrochloride) was prepared and sterilized by filtration. Approximately 1 g of frozen leaf tissue was mixed with 25 ml NIBM (0.001% beta-mercapto ethanol in 1X NIB) and incubated on ice for 15 min. After filtering through a 100 μm filter, the pellet was washed with 15 ml NIBM and filtered again with a 40 μm filter. 2 ml of NIBT (10% Triton X-100 in 1X NIB) were added to the 40 ml solution and incubated on ice for 15 min. After centrifugation at 2400 g for 15 min at 6 °C, the pellet was resuspended on ice and 25-35 ml of 1X NIB were added. After another centrifugation at 2400 g for 15 min at 6 °C, the supernatant was removed, and the nuclei pellet was kept on ice. Nuclei were counted after mixing 20 μl Trypan Blue with 10 μl 1X NIB and 10 μl nuclei suspension and loading 10 μl of the resulting mix on a hemocytometer. After counting, 40,000 nuclei were mixed with 1X Tagment DNA (TD) Buffer (Illumina) and 2.5 μl Tagment DNA Enzyme 1 (TDE1) (Illumina) in a 50 μl reaction. After incubation at 37 °C for 30 min, mixing up and down a few times, the tagmented DNA was cleaned-up in 15 μl EB buffer with a MinElute PCR Purification Kit column (Qiagen) and stored at − 20 °C. PCR reaction then was performed after mixing 15 μl of tagmented DNA with 2.5 μl each of 25 μM i5 (AATGATACGGCGACCACCGAGATCTACACNNNNNNNNTCGTCGGCAGCGTC) and i7 (CAAGCAGAAGACGGCATACGAGATNNNNNNNNGTCTCGTGGGCTCGG) PCR primers (where N indicates the presence of an 8-bp barcode) in 1X Q5 HotStart Master Mix (NEB), and incubating 5 min at 72 °C, 30s at 98 °C, followed by 5 cycles of: 10s at 98 °C; 30s at 63 °C; 1 min at 72 °C, and hold at 4 °C. After this initial PCR, 5 μl of amplified DNA was quantified via qPCR (using the same PCR primers) to determine the optimum number of PCR cycles for the remaining 45 μl before over-amplifying the sample (characterized by ¼ of the maximum fluorescent intensity on the qPCR plot). Typically, PCR reactions were completed after adding 6–8 amplification cycles. The DNA then was cleaned-up with a MinElute column and resuspended in 15 μl EB buffer (Qiagen).

### Sequencing and data analysis

Sequencing of the two Hi-C biological replicates (replicates 1 and 2) was performed on Illumina HiSeq 2500 and NovaSeq 6000 systems, at 2x150bps. Filtering of the raw read data and mapping to the B73 AGPv4 reference genome sequence were performed using the Juicer package [17]. Frequency of contacts were plotted on a 2D matrix using the Juicebox utility [18]. Chromosomal A/B compartments predictions for chromosome 1 were made using Juicer-Eigenvector at bin sizes 250 and 500 Kbps. Hierarchical TADs were called for both Hi-C replicates using HiTAD from the TADLib package [14]. Default parameters were used. The related runHiC pipeline was run for each replicate, also with defaults, to generate the .cool files needed for HiTAD analysis. Chromatin loops were detected using the HICCUPS software package [4] (from the Juicer package versions 1.9.9 and 1.14.08, for replicates 1 and 2, respectively). Default values for chromatin loop anchor lengths vary from 5 to 25Kbps, with a majority at 10Kbps.

For repeat density analysis, repeat elements for the B73 AGPv4 reference genome sequence were obtained from MaizeGDB. The manner in which these elements overlap the loops predicted from the replicates 1 and 2 data sets was examined. Each loop was treated as the union of two anchor regions and an interior region. These three components were compared to the set of repeat elements using “bedtools -coverage” [19] which reported the number of positions in each covered by at least a single repeat element. With this and the known sizes, the fraction of anchor and interior positions covered in each loop was computed.

For eQTL analysis, 61,188 eQTL records (see Supplemental Table 6 in [13]) provided the genomic location of each eQTL, its lead SNP, and the associated gene relative to the maize B73 RefGen v2 reference. Coordinates of the eQTLs and the SNPs were translated to coordinates in Zm-B73-REFERENCE-GRAMENE-4.0 (AGPv4) using the EnsemblPlants Assembly Converter. Gene names were translated to the B73 v4 Zm00001d.2 gene model set names using cross-reference information provided by MaizeGDB. A number of eQTLs could not be translated directly, as, for example, when the associated gene in RefGen v2 no longer existed in AGPv4 or the converter split it into several segments. In the former case the original record was ignored; in the latter, the segment containing the SNP was retained as the AGPv4 eQTL. Each AGPv4 eQTL record (eQTL region, lead SNP, associated gene) then was examined in conjunction with loop structures and coordinates predicted from replicate 2.

Gramene gene models for build 4.0 of B73 were obtained from the MaizeGDB repository, specifically from the GFF3 file Zm-B73-REFERENCE-GRAMENE-4.0_Zm00001d.2.gff3. Feature types “gene,” “lincRNA_gene,” “miRNA_gene,” and “tRNA_gene” were selected, yielding 44,474 genes. Of these, 38,847 were reported to be associated with chromosomes 1–10 and represent the set used for genic analyses.

RNA-Seq libraries derived from all four biological replicates were sequenced on an Illumina HiSeq 2500 system at 1x50bps (single 3′ end sequenced), targeting ~20MM single reads per replicate. Read sequences were trimmed based on quality scores and those matching B73 mitochondrial, chloroplast, and rRNA sequences were filtered with FACS [20]. The remaining reads were quantified using Kallisto [21] pseudoalignment to the B73 AGPv4 gene transcripts and estimated counts summarized at the gene level. The Sleuth expression analysis suite in R [22] was used to normalize and report the abundances as Transcripts Per Million, or TPM [22]. Individual expression levels (TPM counts) reported for each gene in the four biological replicates were averaged to provide a global average expression level for each gene. Genes with average leaf expression level greater than or equal to one were taken as expressing. With this criterion, 18,700 genes associated with chromosomes 1–10 were listed as “expressed genes” while the remaining 20,147 were listed as “unexpressed”. Finally, genes were mapped to Hi-C chromatin loops using BEDTools [19].

Sequencing of three ATAC-Seq biological replicates was performed on an Illumina NovaSeq 6000 system at 2x50bps. After removing contaminant adapter and organellar sequences, reads were mapped to the AGPv4 reference genome. After filtering for proper read pair alignments and removing duplicated reads, resulting BAM files were separated based on the distance between read pairs into “NF” (“nucleosome-free”) regions and “MN” (“multi-nucleosome”) regions. Reads mapping to specific regions known to exhibit high coverage depth (for example, homologs to organellar genes) were removed using “bedtools -intersect” [19]. NF and MN peaks were called separately with the MACS2 software tool [23]. Consensus peaks were generated by searching for physical overlap (> 1 bp) between peaks generated for each replicate.

ATAC-Seq NF consensus peaks were classified in relation to their proximity to a particular expressing gene. The location of each expressing gene was compared to the set of 19,532 NF consensus peaks on chromosomes 1–10. For each, the closest peak and its distance in bp was determined using “bedtools -closest” [19], comparing the genomic coordinates (start-end) of the gene with the genomic coordinates (start-end) of each peak. Genomic coordinates and strand information for genes were taken from the Gramene gene models GFF3 file. The nearest peak was declared “overlapping” if any part of its span overlapped (at least 1 bp) any part of the gene’s span, in which case “bedtools -closest” reported a genomic distance of 0 bp. Otherwise, a signed distance was reported, negative if the peak was located “left” of the gene, positive when it was located “right” of it. The distance indicated the smallest absolute difference between the endpoints of the gene and the peak. This signed distance and the known strand of the gene allowed determination of the peak as lying 5′ or 3′ of the gene. Peaks then were mapped to gene models using BEDTools [19] according to the following four categories: 1) peaks overlapping a particular gene model; 2) peaks located <2Kbps downstream from a gene model; 3) peaks located <2Kbps upstream from a gene model; and 4) peaks located >2Kbps away from a gene model (upstream or downstream).

Peaks were mapped to chromatin loops using BEDTools [19]. Prioritization of peak overlaps was made necessary by the fact that a large fraction of chromatin loops shared one anchor. It was performed according to the following order: 1) peaks overlapping an anchor region; 2) peaks overlapping a loop interior region; 3) peaks not overlapping any loop regions. Following that order, peaks mapping to category 1 were removed from the list of peaks mapped to category 2 (and from category 3 when mapping to category 2).

## Supplementary Information

**Additional file 1.** Hi-C replicate 1 TAD-like hierarchical domain coordinates. (seqid) Chromosomal assignment for domain and sub-domain; (start0) (end0) Coordinates (in bps) of start and end, respectively, of domain or sub-domain; (level) TAD-like domains are denoted as level 0, while sub-domains are denoted as level 1 or higher.

**Additional file 2.** Hi-C replicate 2 TAD-like hierarchical domain coordinates. Categories are the same as in Additional File 1.

**Additional file 3.** Hi-C replicate 1 chromatin loop coordinates. Coordinates (in bps) and chromosome assignments are shown for both anchors forming a loop. (chr1) Chromosome assignment of first anchor; (× 1, × 2) Coordinates in bps of first anchor; (chr2) Chromosome assignment of second anchor; (y1, y2) Coordinates in bp of second anchor. All coordinates are against the maize B73 AGPv4 assembly.

**Additional file 4.** Hi-C replicate 2 chromatin loop coordinates. Coordinates (in bps) and chromosome assignments are shown for both anchors forming a loop. (chr1) Chromosome assignment of first anchor; (× 1, × 2) Coordinates in bps of first anchor; (chr2) Chromosome assignment of second anchor; (y1, y2) Coordinates in bp of second anchor. All coordinates are against the maize B73 AGPv4 assembly.

**Additional file 5.** ATAC-Seq libraries. Sequencing yields and peak counts are shown for all three biological replicates. All reads were aligned against the maize B73 AGPv4 assembly. Consensus peaks (NF) were used for the study.

**Additional file 6.** ATAC-Seq consensus NF peak coordinates. Coordinates (in bps) and chromosome assignments are shown for all 19,532 consensus NF peaks. (chr) Chromosome assignment; (× 1, × 2) Coordinates in bps of consensus NF peak borders. All coordinates are against the maize B73 AGPv4 assembly.

**Additional file 7.** gene expression data. RNA-Seq were aligned to the maize AGPv4 reference genome and assigned to Gramene gene models for build 4.0 of B73 from the MaizeGDB repository. Genome coordinates and TPM counts for each gene transcripts are shown.

**Additional file 8.** overlap analysis of Hi-C replicate 1 and 2 chromatin loop anchors against expressed genes and ATAC-Seq consensus peaks. See Additional File 6 for details. Features overlapping with loop anchors are listed and counted for each Hi-C replicate (“expressed gene”: gene with expression level above expected threshold “unexpressed gene”: gene with expression level below expected threshold; “overlapping peak”: open chromatin peak overlapping with expressed gene; “peak <2Kbps upstream”: open chromatin peak located <2Kbps upstream from expressed gene; “peak <2Kbps downstream”: open chromatin peak located <2Kbps downstream from expressed gene; “peaks >2Kbps”: open chromatin peak located >2Kbps from expressed gene).

**Additional file 9.** replicate 1 chromatin loop anchor annotation and counts for each feature listed below. (seqid) Chromosomal assignment for loop anchor; (start) (end) Coordinates (in bps) of start and end, respectively, of chromatin loop anchor; (locus) name and loop assignment of anchor; (bare) presence or absence of any of the features listed below on anchor (0 = at least one feature present; 1 = no feature present). The following features are annotated: (ef) (uf) expressed (ef) or unexpressed (uf) gene with nearest open chromatin peak >2Kbps away from gene; (en3) (un3) expressed (en3) or unexpressed (un3) gene with nearest open chromatin peak <2Kbps downstream from gene; (en5) (un5) expressed (en5) or unexpressed (un5) gene with nearest open chromatin peak <2Kbps upstream from gene; (eo) (uo) expressed (eo) or unexpressed (uo) gene with nearest open chromatin peak overlapping with gene; (mp) open chromatin peak overlapping an anchor; (mpfe) open chromatin peak overlapping an anchor but with the nearest expressed gene >2Kbps away from the peak.

## Data Availability

Hi-C interaction matrices, hierarchical TAD-like domain coordinates and chromatin loop coordinates generated during this study have been deposited in NCBI’s Gene Expression Omnibus [https://www.ncbi.nlm.nih.gov/geo] under GEO Series accession number GSE161294. The Hi-C, RNA-Seq and ATAC-Seq read data are available in NCBI’s Sequence Read Archive repository [https://www.ncbi.nlm.nih.gov/sra] under SRA accession number PRJNA647026.
